# Complete plastome sequence of *Changiostyrax dolichocarpa* (Styracaeae): An endangered (EN) plant species endemic to China

**DOI:** 10.1080/23802359.2018.1511847

**Published:** 2018-10-27

**Authors:** Xiu-Lian Cai, Jian-Hua Wang, Kun-Kun Zhao, Zhi-Xin Zhu, Hua-Feng Wang

**Affiliations:** Hainan Key Laboratory for Sustainable Utilization of Tropical Bioresources, Institute of Tropical Agriculture and Forestry, Hainan University, Haikou, China

**Keywords:** *Changiostyrax dolichocarpa*, plastome, phylogeny, genome structure

## Abstract

*Changiostyrax dolichocarpa* is a critically endangered plant species occurring in central and southeastern China. Although the systematic position of *Changiostyrax* was still unclear, morphological characters, plastid and nSSR evidence supported that *C. dolichocarpa* should be separated from *Sinojackia* (Styracaceae). Here, we report and characterize the complete plastid genome sequence of *C. dolichocarpa* in an effort to provide genomic resources useful for promoting its conservation. The complete plastome is 158,821 bp in length and contains the typical structure and gene content of angiosperm plastome, including two inverted repeat (IR) regions of 26,000 bp, a large single copy (LSC) region of 88,038 bp and a small single copy (SSC) region of 18,784 bp. The plastome contains 120 genes, consisting of 83 unique protein-coding genes, 37 unique tRNA gene, and eight unique rRNA genes. The overall A/T content in the plastome of *C. dolichocarpa* is 62.70%. The complete plastome sequence of *C. dolichocarpa* will provide a useful resource for the conservation genetics of this species as well as for the phylogenetic studies for *Changiostyrax*.

*Changiostyrax dolichocarpa* C.T.Chen (Styracaeae, formerly *Sinojackia dolichocarpa*) is a rare and endangered plant of the family Styracaceae (Qin et al. 2017) and distributed in Hubei and Hunan Province of China (Li et al. 2002), which also has potential horticultural value. Based on the morphological characteristics of the fruit and flower, *C. dolichocarpa* was recognized as a species within the independent genus (*Changiostyrax*) (Chen 1995). Although the systematic position of *Changiostyrax* was still unclear, morphological characters, plastid and nSSR evidence supported that *C. dolichocarpa* should be separated from *Sinojackia* (Styracaceae) (Yao et al. 2008). Consequently, the genetic and genomic information is urgently needed to promote its systematics research and the development of conservation value of *C. dolichocarpa*. Here, we report and characterize the complete plastome of *C. dolichocarpa* (GenBank accession number: this study). This is the first report of a complete plastome for the genus *Changiostyrax.*

In this study, *C. dolichocarpa* was sampled from Hunan Hupingshan National Nature Reserve in Hunan province of China (111.386°E, 29.591°N). A voucher specimen (H.F. Wang, sm2) was deposited in the Herbarium of the Institute of Tropical Agriculture and Forestry (HUTB), Hainan University, Haikou, China.

The modified cetyltrimethyl ammonium bromide (CTAB) protocol of Doyle and Doyle (1987) was used to extract genomic DNA from dry leaf tissues. The genomic DNA of each sample was quantified and analysed with Agilent 2100 BioAnalyzer. Samples yield at least 0.8 μg of DNA which were selected for subsequent libraries construction and *de novo* sequencing. Genomic DNA of selected samples was used to build the paired-end libraries with 200–400 bp insert size. Libraries were sequenced using BGISEQ-500 platform at BGI Shenzhen, China and produced about 8 Gb high quality per sample with 100 bp paired-end reads. Raw reads were trimmed using SOAPfilter_v2.2 with the following criteria: (1) reads with >10% base of *N*; (2) reads with >40% of low quality (value ≤10); (3) reads contaminated by adaptor and produced by PCR duplication. Around 6 Gb clean data were assembled against the plastome of *Sinojackia rehderiana* (MF179499.1) (Yu et al. 2017) using MITO bim v1.8 (Hahn et al. 2013).

The plastome was annotated using Geneious R8.0.2 (Biomatters Ltd., Auckland, New Zealand) against the plastome of *Sinojackia xylocarpa* (NC035418). The annotation was corrected with DOGMA (Wyman et al. 2004). A circular plastome map was generated using OGDRAW (http://ogdraw.mpimp-golm.mpg.de/) (Lohse et al. 2013).

The plastome of *C. dolichocarpa* was found to possess a total length of 158,821 bp with the typical quadripartite structure of angiosperms, containing two inverted repeats (IRs) of 26,000 bp, a large single copy (LSC) region of 88,038 bp and a small single copy (SSC) region of 18,784 bp. The plastome contains 120 genes, consisting of 83 unique protein-coding genes, 37 unique tRNA gene and four unique rRNA genes. Among these gene, 15 genes (*trnK-UUU, trnG-GCC, trnL-UAA, trnV-UAC, trnI-GAU, trnA-UGC, rpl12, rps16, atpF, rpoC1, petB, petD, rpl16, ndhB, ndhA*) possessed a single intron and three genes (*ycf3, clpP, rps12*) had two introns. The gene *rps12* was found to be trans-spliced, as is typical of angiosperms. The overall A/T content in the plastome of *C. dolichocarpa* is 62.70%, in which the corresponding value of the LSC, SSC, and IR region was 64.70%, 69.50%, and 57.00%, respectively.

We used RAxML (Stamatakis 2006) with 1000 bootstraps under the GTRGAMMAI substitution model to reconstruct a maximum likelihood (ML) phylogeny of six published complete plastome of Styracaceae, using *Symplocos ovatilobata* and *Symplocos costaricana* (symplocaceae) outgroups. The phylogenetic analysis indicated that *C. dolichocarpa* is closer to *Melliodendron xylocarpurn* than *Sinojackia spp.* (Figure 1). Most nodes in the plastome ML trees were strongly supported. The complete plastome sequence of *C. dolichocarpa* will provide a useful resource for the conservation genetics of this species as well as for the phylogenetic studies for *Changiostyrax*.

**Figure 1.**
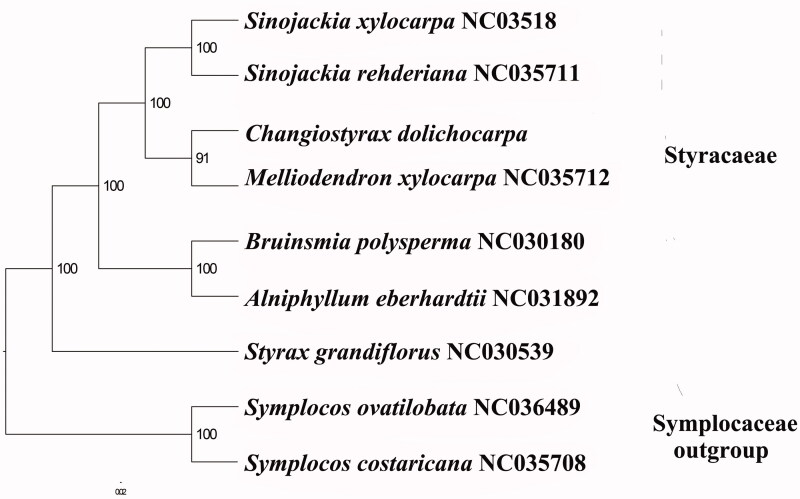
The best ML phylogeny recovered from the combined sequences of the 84 coding genes by RAxML. Accession numbers: *Sinojackia xylocarpa* NC_035418.1, *Sinojackia rehderiana* NC_035711.1, *Changiostyrax dolichocarpa* (this study), MH665364, *Melliodendron xylocarpum* NC_035712.1, *Bruinsmia polysperma* NC_030180.1, *Alniphyllum eberhardtii* NC_031892.1, *Styrax grandiflorus* NC_030539.1, *Symplocos ovatilobata* NC_036489.1, *Symplocos costaricana* NC_035708.1.
